# Diagnostic performance of anthropometric indicators used to assess excess body fat in adolescence

**DOI:** 10.1590/1984-0462/2023/41/2021189

**Published:** 2022-07-06

**Authors:** Nelma Maria Neves Antunes, Marise Fagundes Silveira, Rosângela Ramos Veloso Silva, Josiane Santos Brant Rocha, Fernanda Piana Santos Lima de Oliveira, Sélen Jaqueline Souza Ruas, Fabiana Aparecida Maia Borborema, Jose Henrique Pinto Duarte, Carolina Amaral Oliveira Rodrigues, Maria Fernanda Santos Figueiredo Brito, Antônio Prates Caldeira, Lucinéia de Pinho

**Affiliations:** a Universidade Estadual de Montes Claros, Montes Claros, MG, Brazil.; b Centro Universitário FIPMOC, Montes Claros, MG, Brazil.

**Keywords:** Obesity, Anthropometry, Students, Adolescent, Obesidade, Antropometria, Estudantes, Adolescente

## Abstract

**Objective:**

To compare the performance of anthropometric indicators that identify excess body fat (BF) in adolescents.

**Methods:**

This is a methodological study that used probability cluster sampling through school and class draws. Data collection included sociodemographic characteristics and anthropometric measures. Body mass index (BMI), waist-to-height ratio (WtHR), conicity index (C index), and waist circumference (WC) were calculated. Body fat percentage (BF%) was calculated from skinfold thickness and used as the gold standard. To analyze the data, descriptive statistics, Student’s t-test, Receiver Operating Characteristic (ROC) curve, and Youden’s index were used, in addition to correlation coefficient calculation between the indicators and BF%.

**Results:**

A total of 997 adolescents enrolled in municipal secondary schools participated in the study. By calculating the BMI, we found that 10.6% of adolescents were overweight, and 4.7% were obese. BMI, WC, and WtHR had the highest accuracy to predict body fatness. All the anthropometric indicators had higher specificity than sensitivity to diagnose excess BF in males. WC had the highest sensitivity in both genders. C index had the smallest area under the ROC curve and the lowest sensitivity in both genders, but its specificity was equivalent to that of the other indicators.

**Conclusions:**

BMI, WtHR, and WC were the best anthropometric indicators to predict excess BF in adolescents and had the best correlation coefficients. These tools can be considered in the screening to detect excess BF in adolescents.

## RESUMO

**Objetivo:**

Comparar o desempenho de indicadores antropométricos para identificar o excesso de gordura corporal (GC) em adolescentes.

**Métodos:**

Estudo metodológico com amostragem probabilística por conglomerados, por sorteio das escolas e turmas. A coleta de dados incluiu características sociodemográficas e medidas antropométricas. O índice de massa corporal (IMC), a relação cintura-estatura (RCE), o índice de conicidade (IC) e a circunferência da cintura (CC) foram calculados. O percentual de gordura corporal (%GC) foi calculado com base na espessura das dobras cutâneas e utilizado como padrão-ouro. Para analisar os dados, foram utilizadas estatísticas descritivas, teste *t* de Student, curva *Receiver Operating Characteristic* (ROC) e índice de Youden. Foi calculado o coeficiente de correlação entre os indicadores e o %GC.

**Resultados:**

Participaram do estudo 997 adolescentes de escolas municipais. Identificou-se, pelo IMC, que 10,6% dos adolescentes apresentavam sobrepeso e 4,7%, obesidade. O IMC, a CC e a RCE apresentaram a maior acurácia para predizer a gordura corporal. Todos os indicadores antropométricos apresentaram especificidade superior à sensibilidade para diagnosticar excesso de GC no sexo masculino. A CC apresentou a maior sensibilidade em ambos os sexos. O IC apresentou a menor área sob a curva ROC e a menor sensibilidade em ambos os sexos, mas especificidade equivalente aos demais indicadores.

**Conclusões:**

IMC, RCE e CC apresentaram a melhor capacidade de predizer excesso de GC em adolescentes e os melhores coeficientes de correlação. Essas ferramentas podem ser consideradas formas de rastreamento na identificação do excesso de GC em adolescentes.

## INTRODUCTION

Overweight is important public health problems that affect more than 670 million people worldwide.^1^ Their etiology involves genetic, metabolic, hormonal, environmental, and behavioral aspects, in addition to cultural and social characteristics.^1–3^ An analysis carried out with population-based studies in more than 200 countries found that the proportion of age-standardized mean body mass index (BMI) and the prevalence of overweight and obesity increased worldwide among adolescents from 1975 to 2016.^1^

Overweight, beginning in childhood and maintained during adolescence, is more likely to be maintained and even worsen in adulthood,^2^ accompanied by consequences to health,^2^ such as increased risk of cardiovascular diseases,^2^ type 2 diabetes,^4^ some types of cancer,^4^ dyslipidemias,^5^ and reduced physical, emotional, and social well-being.^3^ Therefore, monitoring and detecting overweight in early adolescence has become fundamental.^5,6^

For these clinical conditions to be diagnosed and prevented, excess body fat needs to be recognized.^7^ In this context, anthropometric indicators have been widely used due to their easy implementation and low cost to assess the nutritional status of the population. Among them, we highlight body fat percentage (BF%), calculated from skinfold thickness,^8^ body mass index (BMI),^4,6,7,9^ waist circumference (WC),^7^ waist-to-height ratio (WtHR),^8^ and the conicity index (CI).^5,10–11^

BF% is a widely used measure to assess excess BF, determined by different methods such as dual-energy X-ray absorptiometry (DEXA).^7,12^ Another method that has been considered an excellent predictor of subcutaneous fat in children and adolescents is the assessment through skinfold thickness.^13,14^

Given the health problems that overweight can cause and as a way to contribute to clinical practice, this study aimed to compare the performance of anthropometric indicators that detect excess BF in adolescent students from a city located in the north region of Minas Gerais, Brazil, as well as assess the prevalence of overweight and determine the cutoff values of excess BF in the study population.

## METHOD

This is a methodological study. The target population was composed of students enrolled in municipal secondary schools of Montes Claros. The sample size was defined as follows: 95% confidence interval (95%CI), estimated prevalence of 30%,^3^ and sampling error of 4%, considering an adolescent population of approximately 20,000 in the study age group, enrolled. For design effect, the number defined by the calculation was multiplied by a correction factor equal to two, considering that this is cluster sampling (deff=2). Thus, the minimum number of students defined for the study was 984 individuals.

The sample was selected by probability cluster sampling. Both the schools and classes were drawn by simple random sampling. All the students of the drawn classes were invited to participate in the research. The inclusion criteria were as follows: a student aged 11–14 years and regularly enrolled in the school and in the selected class. Adolescents without a duly authorized consent form were excluded.

Data were collected in the second semester of 2017 by a previously trained team composed of professionals from the areas of physical education, nutrition, speech therapy, medicine, and nursing, and by undergraduate students with scientific initiation. Data collected were sociodemographic characteristics and anthropometric data. Weight, height, and WC were measured^6^ for the calculation of BMI, WtHR, and C index. The triceps, subscapular, and calf skinfolds were measured to calculate BF%, which was used as the gold standard.^5,14^

Bodyweight was measured using a portable digital electronic Omron® scale (HBF514C, Japan) with a maximum capacity of 150 kg, accurate to 100g. Height was measured using a Sanny® stadiometer (ES2060, Brazil) with a 35.0–213.0cm measuring range, accurate to 0.1cm. During measurement, the adolescents were instructed to keep their feet together, on the center of the equipment, with their heads, buttocks, and heels touching the vertical table of the stadiometer. The stadiometer ruler was then moved to the subject’s head, and the value was read after a normal outbreath.

WC was measured with an inelastic metric tape Sanny (ES2060) during a normal outbreath, using the average distance between the lower edge of the last rib and the upper edge of the iliac crest as the point of reference. Skinfold measurements were obtained by following the protocols recommended in the literature.^8,14^ Cescorf caliper (Cescorf, Brazil) was used always on the right side of the body.

The triceps skinfold was measured on the back of the right arm vertically to the midline, on the triceps muscle, by pinching the skin and subcutaneous tissue between the thumb and index finger. The subscapular skinfold was measured right below the inferior angle of the right scapula, along the skin cleavage line, in an oblique direction. The skin and subcutaneous tissue were pinched, at an angle of 45° from the horizontal plane, heading upward and inward. The calf skinfold was measured at the maximum circumference of the calf, vertically, where the skinfold was pinched, with the student in the sitting position, with his/her knees and hip flexed to 90°.^8^

The procedure was repeated three times at each site, and the average of the measurements was calculated. The BF% was calculated using the equation proposed by Slaughter et al.^14^ for both genders and the classification of Lohman et al.^13^ Reference values for %BF in children and adolescents as suggested by Lohman are as follows: %BF between 10 and 20% for boys and 15 and 25% for girls indicates normality, %BF between 20 and 25% for boys and 25 and 30% for girls indicates overweight, and %BF between 25 and 30% for boys and 30 and 35% for girls indicates obesity.^13^

BMI was calculated by dividing weight in kilograms by height in square meters (BMI=weight/height^2^) (kg/m^2^), and adopted the classifications of eutrophic, overweight, or obese based on the Z score criteria established by the World Health Organization (WHO) to age and gender.^6^

WHtR was calculated by dividing WC (cm) by height (cm) (WHtR=WC/height)^9^ and using a cutoff point of 0.5, indicating that the normal waist value should be less than half the height.^7,9^

To calculate C index, the formula proposed by Valdez^5,11^ was used, in which the numerator is the WC value in meters and the denominator is the constant 0.109 multiplied by the square root of weight (kg) divided by height (cm), according to the Equation 1 below:


(1) $$C\;Index=\frac{Waist\;circunference}{0,109\sqrt{\frac{Body\;weigth\;(kg)}{Heigth\;(m)}}}$$

Before data collection, a pilot study was carried out from a school that had the same characteristics as the schools of the study to standardize research procedures. The team responsible for the research was trained and supervised by a qualified professional to perform weight, height, and WC measurements. All the skinfold measurements were performed by two able professionals.

To evaluate the predictive capacity of BMI, WtHR, WC, and C index in the identification of individuals with excess BF, we performed a receiver operating characteristic (ROC) curve analysis, assuming %BF as the gold standard.^5,14^ The areas under the ROC curves were estimated, using sensitivity and specificity values with their respective 95% confidence intervals. The critical values (cutoff points) of the curves for overweigh detection were determined using Youden’s index^15^ [(sensitivity+specificity)-1)]. Correlation coefficients between %BF and anthropometric indicators were calculated. All the analyses were performed for each sex, with a 5% significance level. The *Statistical Package for the Social Sciences* (SPSS) software, version 23.0, and MedCalc version 19.1.7 were used for the calculations.

The area under the curve (AUC) indicates the global probability of a test, correctly classifying the presence or absence of a given event. AUC-ROC was classified as follows: 1.00 means a perfect test, values ranging from 0.90 to 0.99 indicate an excellent test, values from 0.80 to 0.89 indicate a good test, values from 0.70 to 0.79 indicate a fair test, values from 0.60 to 0.69 indicate a poor test, and values from 0.50 to 0.59 indicate a useless test.^16^

To assess the correlation between %BF and the other anthropometric indicators, scatter diagrams were plotted, and the Pearson’s linear correlation coefficient was calculated.

The study complies with Resolution 466/12 and was approved by the Research Ethics Committee of Universidade Estadual de Montes Claros under number 1.908.982. The students who participated in the study were those who signed an informed assent form and whose parents or guardians signed a consent form as well.

## RESULTS

A total of 997 adolescent students participated in the study, with a predominance of the female sex (52.8%). The mean age of participants was 12.6 years for the female sex and 12.7 years for the male sex (standard deviation=±0.12).

The assessment identified excess weight in 143 (15.3%) students; 99 (10.6%) students were overweight and 44 (4.7%) were obese, calculated using the prevalence of BMI. We found that excess weight was more frequent among boys (16.5%) compared with girls (14.3%).

BMI, WtHR, WC, and C index had high specificity values to diagnose high BF in the male sex, above 80% (Table 1). In the female sex, WC had the lowest specificity, 67.14% (95%CI 60.4–73.4), based on the cutoff points identified in the ROC curve. As for sensitivity, the values ranged from 33.6% (95%CI 27.1–40.8) to 70.0% (95%CI 63.3–76.2) in the male sex, while in the female sex, they ranged from 36.8% (95%CI 31.0–42.9) to 87.0% (95%CI 82.5–90.7), depending on the anthropometric indicator. The lowest sensitivity values referred to C index for both genders (Table 1). WC had the highest sensitivity value in both genders: 70.0% (95%CI 63.3–76.2) for the male sex and 87.0% (95%CI 82.5–90.7) for the female sex.

**Table 1:** Diagnostic properties of the overweight/obesity anthropometric indicators to detect high body fat percentage in adolescents according to sex (*Receiver Operating Characteristics Curve*).

Anthropometric indicators		Area under the curve (95%CI)	Cutoff	Sensitivity % (95%CI)	Specificity% (95%CI)
Males					
	BMI	0.85 (0.82–0.89)	>19.7	68.3 (61.4–74.8)	89.1 (84.5–92.8)
	WtHR	0.83 (0.79–0.86)	>0.42	69.3 (62.4–75.8)	85.6 (80.5–89.8)
	WC	0.80 (0.77–0.84)	>66	70.0 (63.3–76.2)	80.3 (74.8–85.1)
	Cindex	0.62 (0.57–0.66)	>1.14	33.6 (27.1–40.8)	88.1 (83.3–92.0)
Females					
	BMI	0.89 (0.85–0.91)	>19.4	75.0 (69.5–80.1)	86.6 (81.2–90.9)
	WtHR	0.82 (0.78–0.85)	>0.40	72.4 (66.7–77.7)	79.4 (73.3–84.7)
	WC	0.85 (0.81–0.87)	>61	87.0 (82.5–90.7)	67.1 (60.4–73.4)
	Cindex	0.56 (0.51–0.60)	>1.09	36.8 (31.0–42.9)	75.1 (68.7–80.8)

95%CI: 95% confidence interval; BMI: body mass index; WC: waist circumference; WtHR: waist-to-height ratio; C index: conicity index.

In the evaluation of the AUC-ROC, the C index had the smallest AUC, equal to 0.62 (95%CI 0.57–0.66) for the male sex and 0.56 (95%CI 0.51–0.60) for the female sex, values considered to be low, indicating a low discriminatory power to predict excess BF (p≤0.01). The AUCs for BMI, WtHR, and WC were 0.85 (C index=0.82–0.89), 0.83 (C index=0.79–0.86), and 0.80 (C index=0.77–0.84), respectively (Table 1).

**Figure 1:**
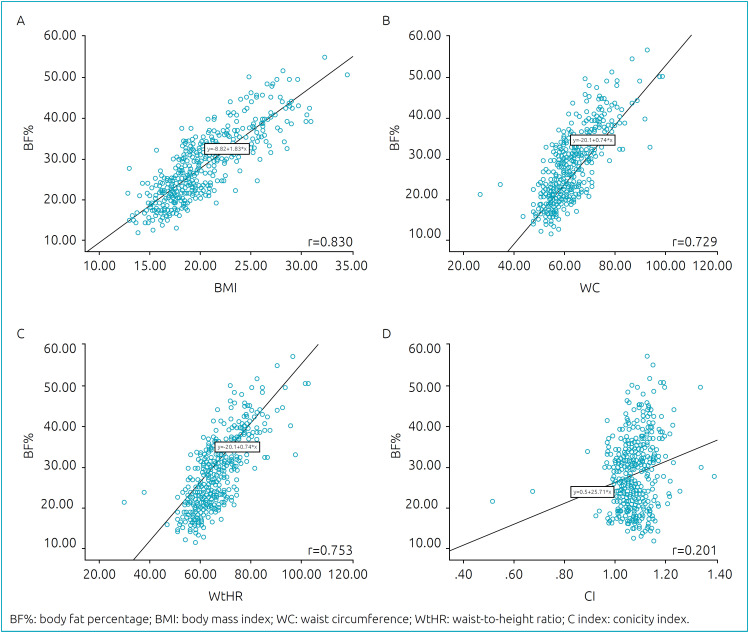
Scatterplot for the variables BF% and body mass index (A), waist circumference (B), waist-to-height ratio (C), and C index (D) for female adolescents.

**Figure 2:**
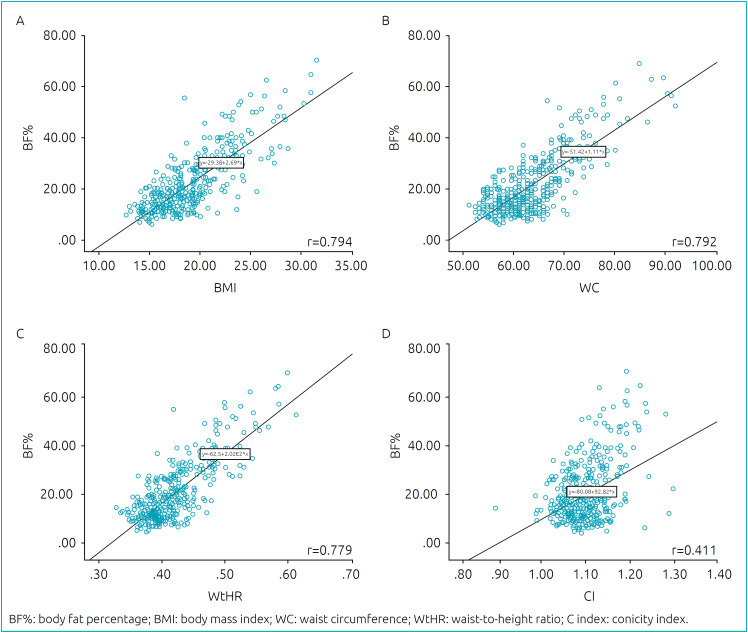
Scatterplot for the variables BF% and body mass index (A), waist circumference (B), waist-to-height ratio (C), and C index (D) for male adolescents.

When comparing BF% with BMI, WC, WtHR, and C index among female adolescents, most measures showed a strong correlation, except for the C index, which had a coefficient equal to 0.20, as shown in Figure 1. When comparing male adolescents, it was again noted that the measures were strongly correlated (r˃0.7), except for the C index (r=0.41; Figure 2).

## DISCUSSION

The study identified the prevalence of overweight in adolescents and the diagnostic properties of the anthropometric indicators of obesity for the detection of high %GC according to sex, assessed through the ROC curve, in addition to providing the correlations between %GC and indicators.

The results of this study referring to the prevalence of overweight were similar to the results of other surveys carried out with adolescents in Bogotá and China.^17,18^ However, higher prevalence was found in a study conducted in Spain, where a research study involving 7,438 teenagers found a 24.9% prevalence of overweight and an 8.2% prevalence of obesity.^19^

A study developed with adolescents in the state of São Paulo found that 18.2% were overweight and 12.7% were obese.^20^ A study carried out in 2014, also in Montes Claros, recorded a 18.5% prevalence of overweight in 535 adolescents from municipal schools.^21^ In addition, higher prevalence values of overweight were observed in adolescents of the great Brazilian regions.^3,22^ The discrepant prevalence of excess weight values may derive from a lack of standardization of the age groups involved in each study and from the methodology employed.

The ROC curve has been used in many epidemiological. This type of analysis identifies the optimal cutoff point and highlights the AUC, which translates the discriminatory power of an indicator for a given outcome.^23,24^

BMI, WHtR, and WC were the indicators that showed the largest areas under the ROC curve, above 0.80, with no significant difference between them, and the accuracy in discriminating BF in both genders was considered to be good. C index, in turn, with the smallest area under the curve, was classified as poor.^15^ A systematic review and meta-analysis, which included cross-sectional and clinical studies with teenagers, showed that the anthropometric indicators that had high discriminatory power to identify high BF were BMI, WC, and WtHR.^25^ A North American survey of adolescents found that BMI, WC, WtHR, and triceps skinfolds can effectively predict adiposity.^26^

These results showed that the anthropometric measurements analyzed can be used to assess the amount and distribution of BF and identify excess BF in adolescents. These indicators have been considered useful measures to predict insulin resistance,^27^ high blood pressure, and metabolic syndrome^24^ and to help assess the prevalence of cardiovascular disease risk in this population.^24^

The BMI cutoff point was 19.7 for boys and 19.4 for girls to identify excess BF. With these cutoff points, it was possible to note that BMI had higher specificity than sensitivity in identifying excess BF for both genders.

A study carried out with adolescents in the Northeastern of Brazil found the following cutoff points for BMI: 22.9 for boys and 22.7 for girls.^24^ In another national study, the cutoff point was 21.0 for boys and 19.5 for girls to assess overweight/obesity related to eating and behavioral factors.^20^ A possible justification for the different cutoff points found can be the existing methodological differences.

The cutoff points in different studies^20,24^ were higher for boys compared with girls, which is consistent with our research findings. BMI is relevant in epidemiological studies for its simplicity, for allowing the classification of the anthropometric status, and for monitoring excess weight in the population. Despite being a measure of general obesity,^4,7^ it showed a good correlation with high %BF.

Regarding WHtR measurement to predict the risk of high %BF, it was observed that the cutoff point was 0.42 for boys and 0.40 for girls to detect high %BF. The findings show that, with these cutoff points, WtHR had higher specificity than sensitivity to diagnose high %BF in both genders. Equivalent results were found in a study conducted on teenagers from Santa Catarina.^5^ Out of adolescents in Piauí, the cutoff points were 0.45 and 0.44 for boys and girls, respectively.^16^ All of these cutoff points are lower than 0.5 proposed for the general population.^8^

WtHR is an additional index to assess obesity and central adiposity, favoring the early detection of adolescents at nutritional risk.^8^ It has been shown that it has a good correlation with visceral fat and is also a predictor of cardiometabolic risk.^7^

As for the use of WC, the best cutoff point to detect high %BF was 66 for boys and 61cm for girls. With these cutoff points, the indicator had the highest sensitivity, in both genders, to identify high %BF. The cutoff points from other studies performed with adolescents ranged from 66 to 67.7cm for girls and 71 to 75.7 for boys.^5,20^ These values are higher than the ones found in our study. Both studies included older adolescents, which might explain the differences.

WC is a measure that has been used to diagnose central obesity. It is related to metabolic risk, and, in obese adolescents, it has shown a correlation with abdominal fat and metabolic alterations.^7^

The C index is a parameter that also assesses obesity and BF distribution.^5,10^ It was observed in this study that the best IC cutoff point was 1.14 in boys and 1.09 in girls to identify high %BF, indicating low sensitivity in the diagnosis of excess body adiposity in both sexes, which may result in a high number of false-negative results.

Higher C index cutoff points were found in another Brazilian study carried out with adolescents, being 1.20 for girls and 1.21 for boys.^24^ Another national study carried out with adolescents established as cutoff points the values of 1.14 for girls and 1.13 for boys to assess the association between C index and lipid profile.^10^

The cutoff points of all the anthropometric indicators were higher for the male sex when compared with the female sex, a result that was also found in other studies, regardless of the age group.^5,16,20,24–27^

The measurement of central adiposity is subject to measurement errors, even when performed under protocols and by trained examiners.^28^ One study found that the measurement of general obesity had a lower probability of measurement errors than the measurement of central obesity.^29^ In this study, all assessments were performed in the morning, following the protocols, in an attempt to minimize errors.

It should be noted that when isolated anthropometric measures are used, they have limitations when it comes to screening obesity in adolescents. This study points out these limitations precisely, highlighting opportunities for serial assessments, which can be conducted even in a school environment.

It is important to highlight that the results obtained in this study help assess overweight and obesity among adolescents in the age group of the study sample, as they corroborate the use of simple, easy-to-use, low-cost anthropometric indicators that can be applied to a large number of individuals and in different places, especially in places with lower financial and technological resources and greater social vulnerability. They contribute to overweight and obesity screening efforts and help implement preventive and therapeutic measures in a timely manner among young people and their families.^27^

Our results should be assessed considering some limitations. The first one refers to the use of a double indirect measure to assess BF% as the gold standard for comparison. This was chosen because of operational difficulties and the high cost of measures that are considered more accurate, like DEXA. However, a good correlation has been found between BF% measured through skinfold thickness and measures obtained through indirect methods like DEXA.^12^

Among the indicators analyzed, BMI, WtHR, and WC had similar diagnostic capacities to predict excess BF in adolescents, while C index showed unsatisfactory results in the study population, concerning sensitivity and accuracy to diagnose overweight and obesity. The cutoff points serve only as a reference and not as a definitive diagnosis.

Based on the results found in this study, we conclude that the anthropometric indicators BMI, WtHR, and WC are simple, noninvasive methods that can accurately predict excess BF. Therefore, these indicators can be used to more efficiently monitor the health status of the population.
